# 2-[(1*E*)-[(*Z*)-2-({[(1*Z*)-[(*E*)-2-[(2-Hy­droxy­phen­yl)methyl­idene]hydrazin-1-yl­idene]({[(4-methyl­phen­yl)meth­yl]sulfan­yl})meth­yl]disulfan­yl}({[(4-methyl­phen­yl)meth­yl]sulfan­yl})methyl­idene)hydrazin-1-yl­idene]meth­yl]phenol: crystal structure, Hirshfeld surface analysis and computational study

**DOI:** 10.1107/S2056989020008762

**Published:** 2020-07-10

**Authors:** Georgiana Paulus, Huey Chong Kwong, Karen A. Crouse, Edward R. T. Tiekink

**Affiliations:** a Department of Chemistry, Faculty of Science, Universiti Putra Malaysia, UPM, Serdang 43400, Malaysia; b Research Centre for Crystalline Materials, School of Science and Technology, Sunway University, 47500 Bandar Sunway, Selangor Darul Ehsan, Malaysia

**Keywords:** crystal structure, Schiff base, hydrazine carbodi­thio­ate, hydrogen bonding, Hirshfeld surface analysis, DFT

## Abstract

The complete mol­ecule of the title hydrazine carbodi­thio­ate derivative, C_32_H_30_N_4_O_2_S_4_, is generated by a crystallographic twofold axis that bis­ects the di­sulfide bond. The mol­ecule is twisted about this bond with the C—S—S—C torsion angle of 90.70 (8)° indicating an orthogonal relationship between the symmetry-related halves of the mol­ecule. The conformation about the imine bond [1.282 (2) Å] is *E* and there is limited delocalization of π-electron density over the CN_2_C residue as there is a twist about the N—N bond [C—N—N—C torsion angle = −166.57 (15)°]. An intra­molecular hydroxyl-O—H⋯N(imine) hydrogen bond closes an *S*(6) loop. In the crystal, methyl­ene-C—H⋯π(tol­yl) contacts assemble mol­ecules into a supra­molecular layer propagating in the *ab* plane: the layers stack without directional inter­actions between them. The analysis of the calculated Hirshfeld surfaces confirm the importance of H⋯H contacts, which contribute 46.7% of all contacts followed by H⋯C/C⋯H contacts [25.5%] reflecting, in part, the C—H⋯π(tol­yl) contacts. The calculation of the inter­action energies confirm the importance of the dispersion term and the influence of the stabilizing H⋯H contacts in the inter-layer region.

## Abstract 2

The title hydrazine carbodi­thio­ate derivative is highly twisted as seen in the C—S—S—C torsion angle of 90.70 (8)°; the mol­ecule is twofold symmetric. In the mol­ecular packing, mol­ecules are assembled into supra­molecular layers in the *ab* plane by methyl­ene-C—H⋯π(tol­yl) inter­actions.

## Chemical context   

Schiff base mol­ecules can be derived from the condensation of *S*-alkyl-di­thio­carbazate derivatives with heterocyclic aldehydes and ketones to form mol­ecules of the general formula *R*SC(=S)N(H)N=C(*R*′)*R*′′, where *R*′, *R*′′ = alkyl and aryl. These mol­ecules are effective ligands for a variety of metals and the motivation for complexation largely stems from the promising biological activity exhibited by the derived metal complexes (Low *et al.*, 2016 ▸; Ravoof *et al.*, 2017 ▸; Yusof *et al.*, 2020 ▸). However, these Schiff bases are susceptible to oxidation resulting in the formation of a di­sulfide bond, as has been observed previously (Amirnasr *et al.*, 2014 ▸; Sohtun *et al.*, 2018 ▸). This is the case in the present report where the title compound, (I), was the side-product from the synthesis of the Schiff base, 4-methyl­benzyl-2-(2-hy­droxy­benzyl­idene) hydra­zinecarbodi­thio­ate (Ravoof *et al.*, 2010 ▸). After crystals of the desired Schiff base that had precipitated overnight were removed by filtration, the slow evaporation of the filtrate over a period of several days yielded crystals of (I). Herein, the crystal and mol­ecular structures of (I) are described along with an analysis of the calculated Hirshfeld surfaces and computation of inter­action energies in the crystal.
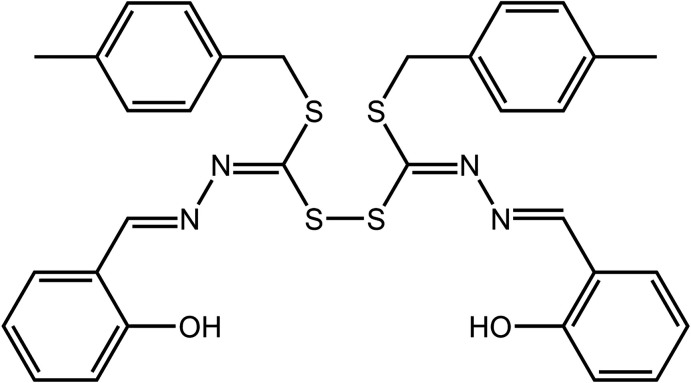


## Structural commentary   

**Figure 1:**
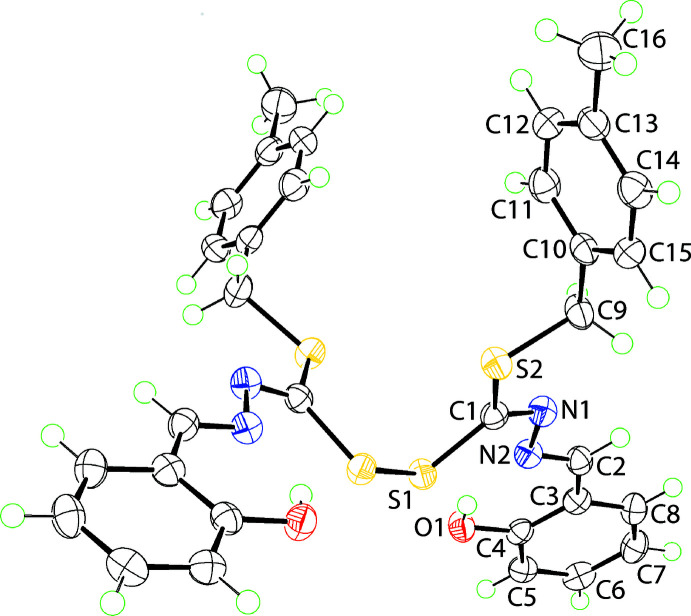
The mol­ecular structure of (I) showing the atom-labelling scheme and displacement ellipsoids at the 70% probability level.

The crystallographic asymmetric unit of (I) comprises half a mol­ecule as it is disposed about a twofold axis of symmetry bis­ecting the di­sulfide bond, Fig. 1 ▸. The C1, N1, S1 and S2 atoms lie in a plane with an r.m.s. deviation of 0.0020 Å. The appended N2 and C5 atoms lie 0.036 (2) and 0.052 (2) Å to one side of the plane and the S1^i^ atom −0.1659 (16) Å to the other side; symmetry operation (i): 1 − *x*, *y*, 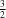 − *z*. The C1—S1 bond length of 1.7921 (17) Å is significantly longer than the C1—S2 bond of 1.7463 (17) Å, which is ascribed to the S1 atom participating in the S1—S1^i^ bond of 2.0439 (8) Å; each C1—S bond is shorter than the C9—S2 bond length of 1.8308 (18) Å.

The sequence of C1=N1 (*E*-conformation), N1—N2 and C2=N2 bond lengths is 1.282 (2), 1.409 (2) and 1.286 (2) Å, respectively, and suggests limited delocalization of π-electron density over this residue which is consistent with a twist about the N1—N2 bond as seen in the C1—N1—N2—C2 torsion angle of −166.57 (15)°. The presence of an intra­molecular hydroxyl-O—H⋯N(imine) hydrogen bond, Table 1 ▸, is noted and accounts for the planarity in this region of the mol­ecule as seen in the values of the N2—C2—C3—C4 and C2—C3—C4—O1 torsion angles of 3.8 (3) and 1.8 (3)°, respectively. The dihedral angle between the hy­droxy­benzene and tolyl rings is 65.11 (6)°, indicating a significant twist in this part of the mol­ecule. Overall, the mol­ecule is twisted about the central di­sulfide bond with the C1—S1—S1^i^—C1^i^ torsion angle being 90.70 (8)° and the dihedral angle between the two CNS_2_ planes being 88.22 (3)°.

## Supra­molecular features   

**Figure 2:**
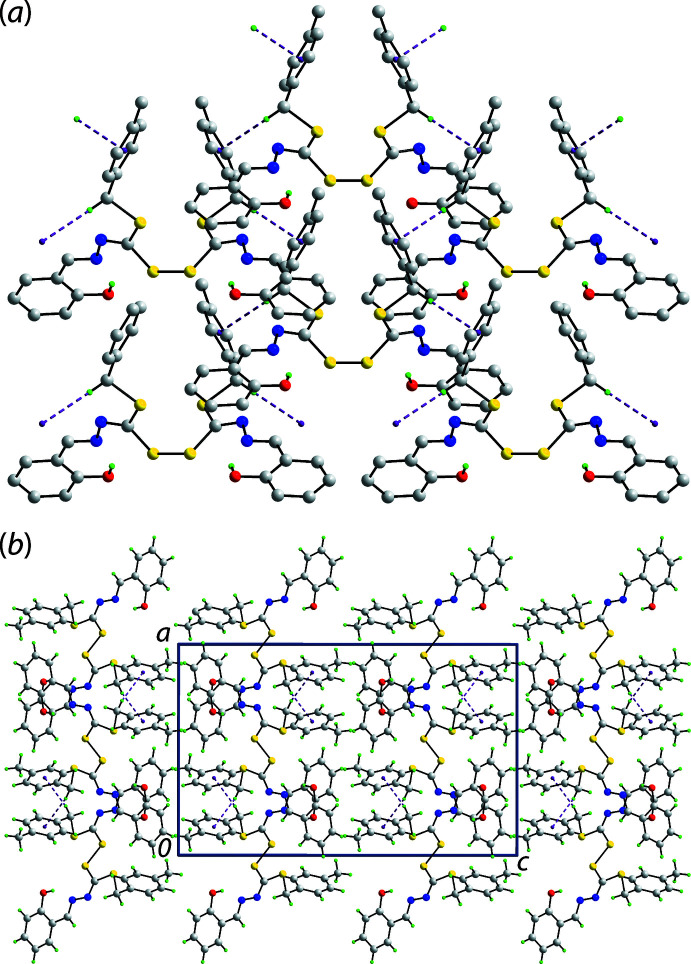
Mol­ecular packing in (I): (*a*) the supra­molecular layer in the *ab* plane sustained by methyl­ene-C—H⋯π(tol­yl) inter­actions shown as purple dashed lines (the non-participating H atoms removed for clarity) and (*b*) a view of the unit-cell contents shown in projection down the *b* axis highlighting the stacking of layers.

In the crystal, the only directional contact identified in the geometric analysis of the mol­ecular packing employing *PLATON* (Spek, 2020 ▸), is a methyl­ene-C—H⋯π(tol­yl) contact, Table 1 ▸. As each mol­ecule donates and accepts two such contacts and these extend laterally, a supra­molecular layer in the *ab* plane is formed, Fig. 2 ▸(*a*). Layers stack along the *c* axis without directional inter­actions between them, Fig. 2 ▸(*b*).

## Analysis of the Hirshfeld surfaces   

**Figure 3:**
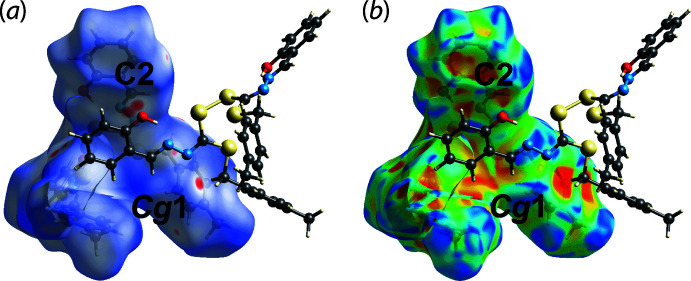
Views of the Hirshfeld surface for (I) mapped over (*a*) *d*_norm_ in the range −0.104 to + 1.517 arbitrary units and (*b*) the shape-index property.

The Hirshfeld surface analysis comprising *d*_norm_ surface, electrostatic potential (calculated using wave function at the HF/STO-3 G level of theory) and two-dimensional fingerprint plot calculations were performed for (I) to qu­antify the inter­atomic inter­actions between mol­ecules. This was accomplished using *Crystal Explorer 17* (Turner *et al.*, 2017 ▸) and following established procedures (Tan *et al.*, 2019 ▸). The bright-red spots on the Hirshfeld surface mapped over *d*_norm_ in Fig. 3 ▸(*a*), *i.e.* near the imine-C2 and tolyl ring, centroid designated *Cg*1, correspond to the C2⋯O1, C2⋯C4 short contacts (with separations ∼0.15 Å shorter than the sum of their van der Waals radii, Table 2 ▸) and the methyl­ene-C9—H9*A*⋯π(tol­yl) inter­action, Table 1 ▸. In addition, this methyl­ene-C9—H9*A*⋯π(tol­yl) inter­action shows up as a distinctive orange ‘pothole’ on the shape-index-mapped Hirshfeld surface, Fig. 3 ▸(*b*).

**Figure 4:**
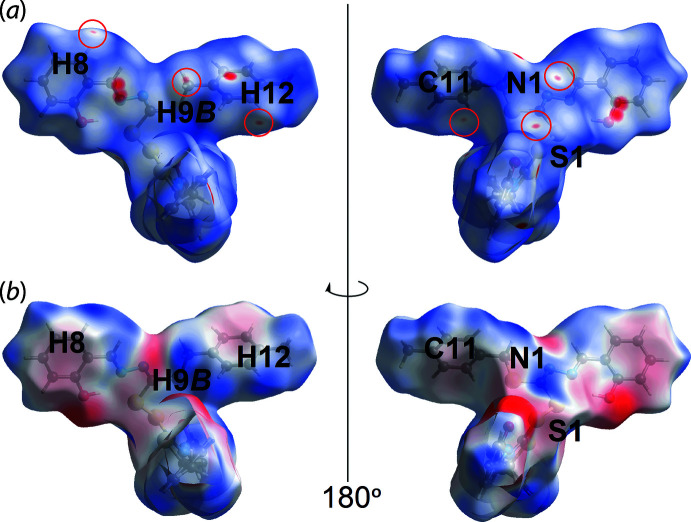
Views of the Hirshfeld surface mapped for (I) over (*a*) *d*_norm_ in the range −0.104 to + 1.517 arbitrary units and (*b*) the calculated electrostatic potential in the range −0.056 to 0.031 a.u. The red and blue regions represent negative and positive electrostatic potentials, respectively.

In the views of Fig. 4 ▸(*a*), the faint red spots appearing near the tolyl-H12, methyl­ene-H9*B* and phenol-H8 atoms correlate with the faint red spots near the sulfanyl-S1, hydrazine-N1 and tolyl-C11 atoms, and correspond to the intra-layer tolyl-C12—H12⋯S1(sulfan­yl), methyl­ene-C9—H9*B*⋯N1(hydrazine) and phenol-C8—H8⋯C11(tol­yl) inter­actions, Table 2 ▸. These inter­actions are also reflected in the Hirshfeld surface mapped over the calculated electrostatic potential in Fig. 4 ▸(*b*), with the blue and red regions corresponding to positive and negative electrostatic potentials, respectively.

**Figure 5:**
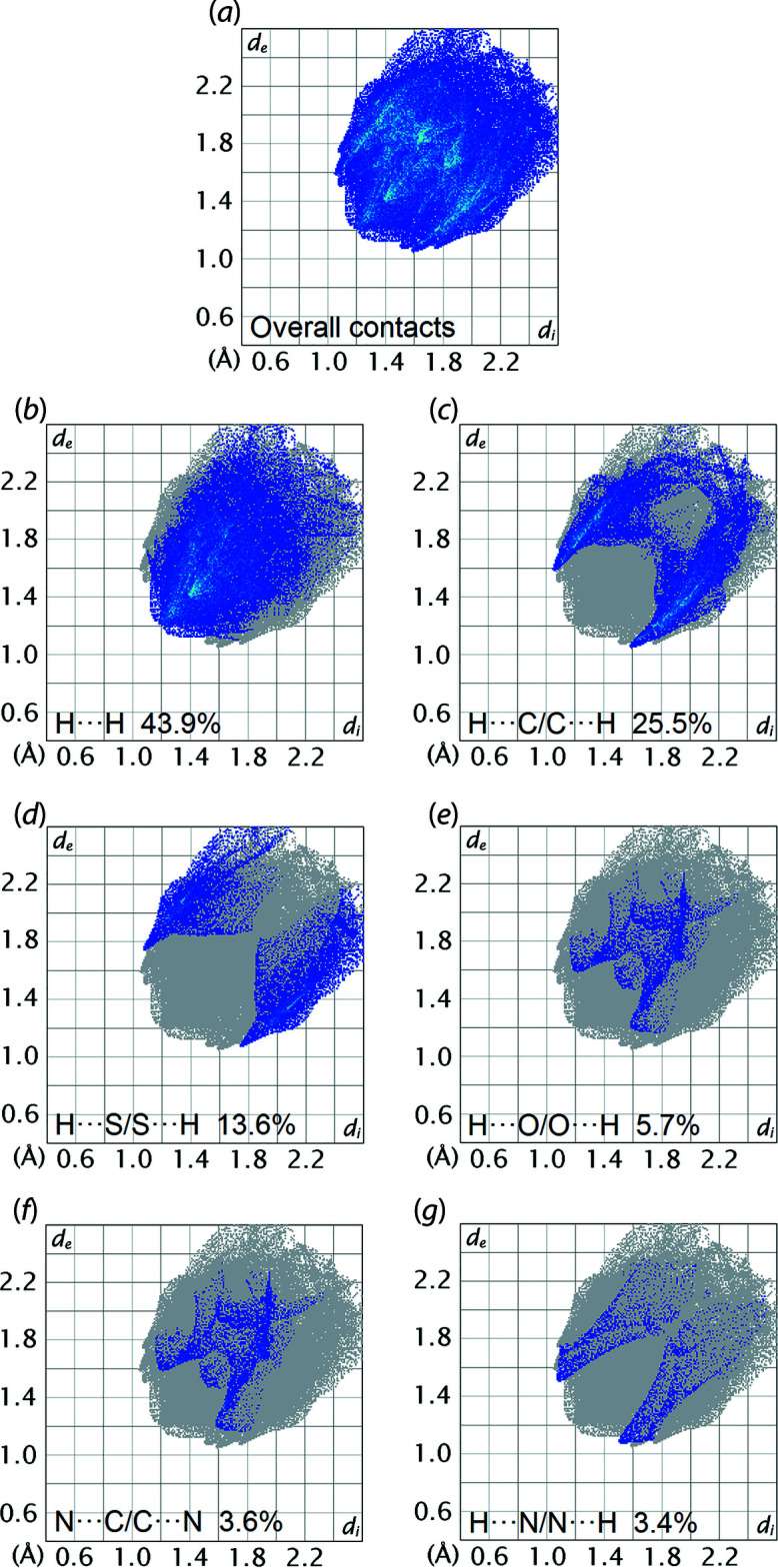
(*a*) A comparison of the full two-dimensional fingerprint plot for (I) and those delineated into (*b*) H⋯H, (*c*) H⋯C/C⋯H, (*d*) H⋯S/S⋯H, (*e*) H⋯O/O⋯H, (*f*) N⋯C/C⋯N and (*g*) H⋯N/N⋯H contacts.

The corresponding two-dimensional fingerprint plots for the calculated Hirshfeld surface of (I) are shown with characteristic pseudo-symmetric wings in the upper left and lower right sides of the *d*_e_ and *d*_i_ diagonal axes for the overall fingerprint plot, Fig. 5 ▸(*a*); those delineated into H⋯H, H⋯C/C⋯H, H⋯S/S⋯H, H⋯O/O⋯H, N⋯C/C⋯N and H⋯N/N⋯H contacts are illustrated in Fig. 5 ▸(*b*)–(*g*), respectively. The percentage contributions for the different inter­atomic contacts to the Hirshfeld surface are summarized in Table 3 ▸. The greatest contribution to the overall Hirshfeld surface is due to H⋯H contacts, which contribute 43.9% and features a round-shaped peak tipped at *d*_e_ = *d*_i_ ∼2.4 Å, Fig. 5 ▸(*b*). The tip of this H⋯H contact corresponds to an inter-layer H6⋯H14 contact with a distance of 2.39 Å, Table 2 ▸; the remaining H⋯H contacts are either around or longer than the sum of their van der Waals radii. The H⋯C/C⋯H contacts contribute 25.5% to the overall Hirshfeld surface, reflecting, in part, the significant C—H⋯π inter­actions evident in the packing, Table 1 ▸. The shortest contacts are reflected as two spikes at *d*_e_ + *d*_i_ ∼2.7 Å in Fig. 5 ▸(*c*). The H⋯S/S⋯H contacts contribute 13.6% and appear as two sharp-symmetric wings at *d*_e_ + *d*_i_ ∼2.8 Å, Fig. 5 ▸(*d*). This feature reflects the intra-layer tolyl-C12—H12⋯S1(sulfan­yl) inter­action, Table 2 ▸. The H⋯O/O⋯H contacts contribute 5.7% and features forceps-like tips at *d*_e_ + *d*_i_ ∼2.8 Å, Fig. 5 ▸(*e*); this separation is ∼0.08 Å longer than the sum of their van der Waals radii. Although both N⋯C/C⋯N and H⋯N/N⋯H contacts appear at *d*_e_ + *d*_i_ ∼2.6–2.8 Å in the respective fingerprint plots, Fig. 5 ▸(*f*) and (*g*), their contributions to the overall Hirshfeld surface are only 3.6 and 3.4%, respectively. The contributions from the other inter­atomic contacts summarized in Table 3 ▸ have an insignificant influence on the calculated Hirshfeld surface of (I).

## Computational chemistry   

In the present analysis, the pairwise inter­action energies between the mol­ecules in the crystal of (I) were calculated by employing the 6-31G(*d*,*p*) basis set with the B3LYP function. The total energy comprises four terms: *i.e*. the electrostatic (*E*_ele_), polarization (*E*_pol_), dispersion (*E*_dis_) and exchange-repulsion (*E*_rep_) energies and these were calculated in *Crystal Explorer 17* (Turner *et al.*, 2017 ▸). The characteristics of the calculated inter­molecular inter­action energies are summarized in Table 4 ▸. As postulated, in the absence of conventional hydrogen bonding in the crystal, the *E*_dis_ energy term makes the major contribution to the inter­action energies. The greatest stabilization energy (–65.7 kJ mol^−1^) occurs within the intra-layer region and arises from the combination of C—H⋯π, C⋯O and C⋯C short contacts as well as weak C—H⋯N/C inter­actions. The second most significant energy of stabilization within the intra-layer region involves a major contribution from the tolyl-C12—H12⋯S1(sulfan­yl) inter­action (dominated by *E*_dis_) with a total energy of −29.7 kJ mol^−1^. In addition, a long-range H6⋯H16*B* contact is observed within the intra-layer region with a H⋯H separation of 2.44 Å.

**Figure 6:**
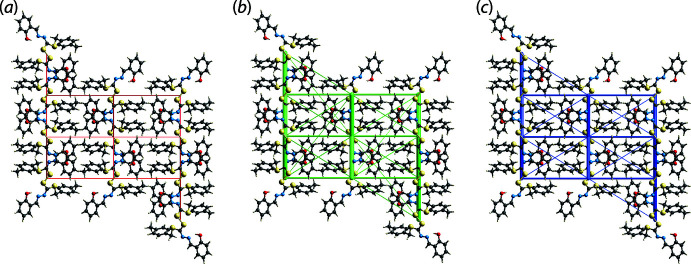
Perspective views of the energy frameworks calculated for (I) showing (*a*) electrostatic potential force, (*b*) dispersion force and (*c*) total energy, each plotted down the *b* axis. The radii of the cylinders are proportional to the relative magnitudes of the corresponding energies and were adjusted to the same scale factor of 55 with a cut-off value of 5 kJ mol^−1^.

The *E*_dis_ energy term also makes the major contribution to the energies of stabilization in the inter-layer region, with the separation between mol­ecules in the inter-layer region being H⋯H contacts. The maximum energy is not found for the shortest H6⋯H14 contact (–9.5 kJ mol^−1^), Table 2 ▸, but rather a pair of phenol-H5⋯H14(tol­yl) contacts (–24.6 kJ mol^−1^), each with a distance of 2.51 Å. Views of the energy framework diagrams down the *b* axis are shown in Fig. 6 ▸ and emphasize the importance of *E*_dis_ in the stabilization of the crystal.

## Database survey   

**Figure 7:**
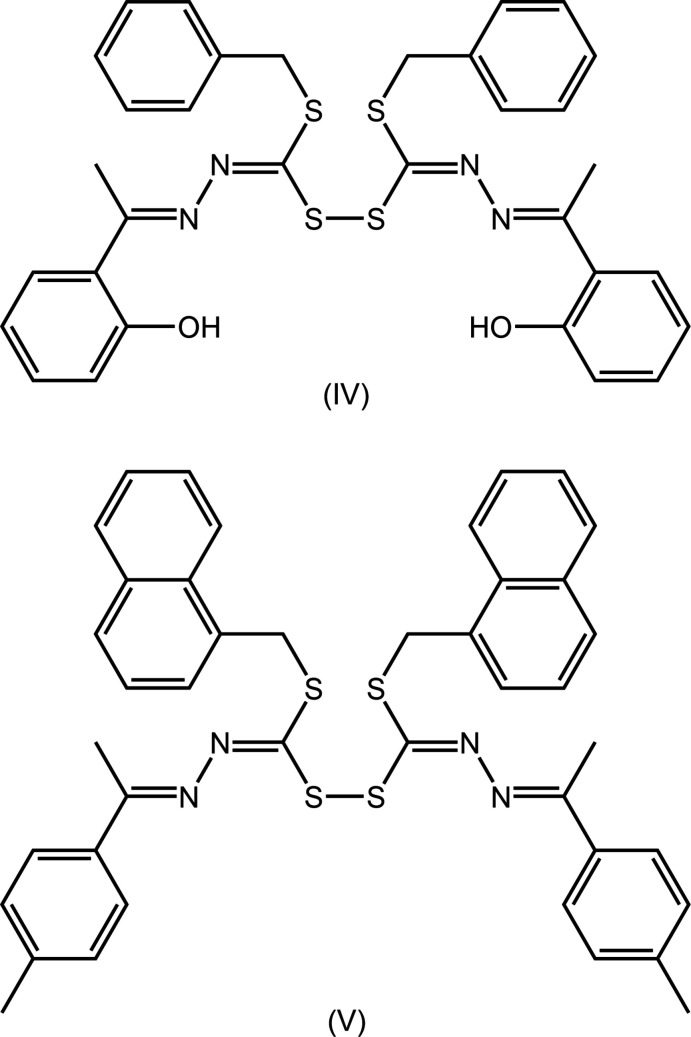
Chemical diagrams for (IV) and (V).

**Figure 8:**
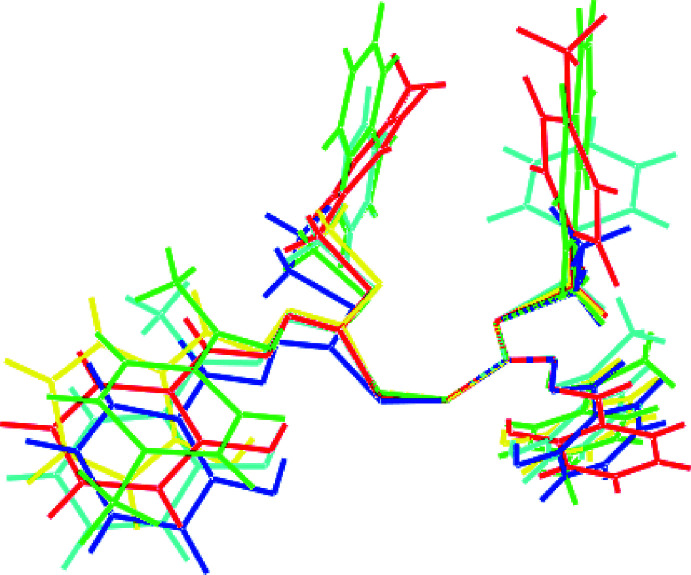
An overlay diagram of (I) red image, (II) yellow, (III) blue, (IV) aqua and (V) green. The mol­ecules have been overlapped so a CS_2_ residue of each mol­ecule is coincident.

In the crystallographic literature, there are four precedents for (I) with details collated in Table 5 ▸. Derivatives (II) and (III) are most closely related to (I), differing only in the nature of the S-bound *R* group, *i.e. R* = Me (MUYRIJ; Madanhire *et al.*, 2015 ▸) and *R* = Et (DIBYOF01; Yekke-ghasemi *et al.*, 2018 ▸), respectively. As shown in Fig. 7 ▸, (IV) is an *S*-benzyl ester with a methyl group on the imine-C atom as well as having the 2-hydroxyl­benzene ring (LAGLUD; Islam *et al.*, 2016 ▸) whereas (V) is an *S*-methyl­naphthyl ester with methyl and 2-tolyl groups bound to the imine-C atom (CUHHET; How *et al.*, 2009 ▸). In common with (I), the complete mol­ecules of (III) and (V) are generated by crystallographically imposed twofold symmetry. While lacking this symmetry, (II) and (IV) approximate twofold symmetry as seen in the overlay diagram of Fig. 8 ▸, from which is observed that to a first approximation, all five mol­ecules adopt a similar conformation. The S—S bond length in (I) lies between the experimentally distinct range of 2.0373 (4) Å in (IV) and 2.0504 (7) Å in (V). In the same way, the C—S—S—C torsion angle in (I) lies between the extreme values of 88.73 (6) and 104.67 (8)° in (II) and (III), respectively.

## Synthesis and crystallization   

Crystals of (I) were isolated from an ethanol–aceto­nitrile solution by slow evaporation and was a side-product from the synthesis of the Schiff base 4-methyl­benzyl-2-(2-hy­droxy­benzyl­idene) hydrazinecarbodi­thio­ate carried out by heating a mixture of *S*-4-methyl­benzyl­dithio­carbazate (10 mmol) and salicyl­aldehyde (10 mmol) in ∼30 ml of aceto­nitrile for about 2 h (Ravoof *et al.*, 2010 ▸). Slow evaporation of the remaining filtrate after removal of the desired product over a period of several days gave yellow plates of (I).

## Refinement   

Crystal data, data collection and structure refinement details are summarized in Table 6 ▸. The carbon-bound H atoms were placed in calculated positions (C—H = 0.95–0.99 Å) and were included in the refinement in the riding-model approximation, with *U*_iso_(H) set to 1.2*U*_eq_(C). The O-bound H atom was located in a difference-Fourier map, but was refined with an O—H = 0.84±0.01 Å distance restraint, and with *U*_iso_(H) set to 1.5*U*_eq_(O).

## Supplementary Material

Crystal structure: contains datablock(s) I, global. DOI: 10.1107/S2056989020008762/hb7929sup1.cif

Structure factors: contains datablock(s) I. DOI: 10.1107/S2056989020008762/hb7929Isup2.hkl

Supporting information file. DOI: 10.1107/S2056989020008762/hb7929Isup3.cml

CCDC reference: 2013050

Additional supporting information:  crystallographic information; 3D view; checkCIF report

## Figures and Tables

**Table 1:** Hydrogen-bond geometry (Å, °) *Cg*1 is the centroid of the (C10–C15) ring.

*D*—H⋯*A*	*D*—H	H⋯*A*	*D*⋯*A*	*D*—H⋯*A*
O1—H1*O*⋯N2	0.84 (2)	1.94 (2)	2.6877 (19)	148 (2)
C9—H9*A*⋯*Cg*1^i^	0.99	2.93	3.9075 (18)	169

Symmetry code: (i) 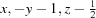.

**Table 2:** A summary of short inter­atomic contacts (Å) for (I)^*a*^

Contact	Distance	Symmetry operation
C2⋯O1	3.07	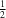 − *x*, 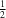 + *y*, *z*
C2⋯C4	3.25	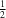 − *x*, 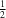 + *y*+, *z*
C12—H12⋯S1	2.82	1 − *x*, 1 + *y*+1, 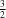 − *z*
C9—H9*B*⋯N1	2.59	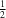 − *x*, 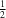 + *y*, *z*
C8—H8⋯C11	2.74	− 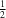 + *x*, − 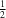 + *y*, 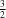 − *z*
H6⋯H14	2.39	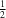 − *x*, 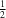 − *y*, 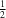 + *z*

Note: (*a*) The inter­atomic distances are calculated in *Crystal Explorer 17* (Turner *et al.*, 2017 ▸) with the *X*—H bond lengths adjusted to their neutron values.

**Table 3:** The percentage contributions of inter­atomic contacts to the Hirshfeld surface for (I)

Contact	Percentage contribution
H⋯H	43.9
H⋯C/C⋯H	25.5
H⋯S/S⋯H	13.6
H⋯O/O⋯H	5.7
N⋯C/C⋯N	3.6
H⋯N/N⋯H	3.4
O⋯C/C⋯O	1.7
C⋯C	1.2
S⋯C/C⋯S	1.0
N⋯N	0.4

**Table 4:** A summary of inter­action energies (kJ mol^−1^) calculated for (I)

Contact	*R* (Å)	*E*_ele_	*E*_pol_	*E*_dis_	*E*_rep_	*E*_tot_
Intra-layer region						
C9—H9*A*⋯*Cg*1^i^ +						
C2⋯O1^ii^ +						
C2⋯C4^ii^ +						
C9—H9*B*⋯N1^ii^ +						
C8—H8⋯C11^iii^	8.70	−19.7	−3.5	−98.9	71.0	−65.7
C12—H12⋯S1^iv^	7.96	−11.1	−1.9	−43.0	33.7	−29.7
H6⋯H16*B* ^v^	14.23	−0.6	−0.2	−6.7	3.0	−4.8
Inter-layer region						
H5⋯H14^vi^	12.44	−10.3	−2.1	−28.1	20.0	−24.6
H16*A*⋯H16*B* ^vii^	15.29	−1.5	−0.4	−11.8	3.5	−10.0
H6⋯H14^viii^	14.93	−3.4	−0.5	−13.6	10.2	−9.5
H6⋯H16*C* ^ix^	15.43	−1.8	−0.4	−10.9	6.2	−7.8
H6⋯H7^*x*^	21.02	−1.2	−0.2	−7.8	5.4	−4.9

Notes: Symmetry operations: (i) −*x* + 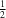, *y* − 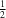, *z*; (ii) −*x* + 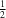, *y* + 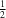, *z*; (iii) *x* − 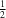, *y* − 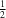, −*z* + 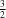; (iv) −*x* + 1, *y* + 1, −*z* + 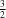; (v) *x* − 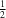, *y* − 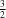, −*z* + 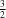; (vi) *x*, −*y* + 1, *z* + 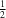; (vii) *x*, −*y* + 2, *z* + 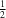; (viii) −*x* + 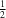, −*y* + 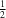, *z* + 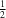; (ix) *x* + 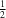, −*y* + 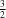, −*z* + 1; (*x*) −*x*, −*y*, −*z* + 2

**Table 5:** A comparison of key geometric parameters (Å, °) in structures related to (I)

Compound	Symmetry	S—S	C—S—S—C	Refcode	Ref.
(I)	2	2.0439 (8)	90.70 (8)	–	This work
(II)	–	2.0386 (7)	88.73 (9)	MUYRIJ	Madanhire *et al.* (2015 ▸)
(III)	2	2.0443 (7)	104.67 (8)	DIBYOF01	Yekke-ghasemi *et al.* (2018 ▸)
(IV)	–	2.0373 (4)	91.54 (6)	LAGLUD	Islam *et al.* (2016 ▸)
(V)	2	2.0504 (7)	96.2 (1)	CUHHET	How *et al.* (2009 ▸)

**Table 6:** Experimental details

Crystal data	
Chemical formula	C_32_H_30_N_4_O_2_S_4_
*M*_r_	630.84
Crystal system, space group	Orthorhombic, *P* *b* *c* *n*
Temperature (K)	100
*a*, *b*, *c* (Å)	15.4653 (4), 7.9639 (2), 24.8116 (7)
*V* (Å^3^)	3055.90 (14)
*Z*	4
Radiation type	Cu *K*α
μ (mm^−1^)	3.15
Crystal size (mm)	0.27 × 0.14 × 0.07

Data collection	
Diffractometer	Agilent Xcalibur, Eos, Gemini
Absorption correction	Multi-scan (*CrysAlis PRO*; Agilent, 2012 ▸)
*T*_min_, *T*_max_	0.819, 1.000
No. of measured, independent and observed [*I* > 2σ(*I*)] reflections	10206, 2933, 2637
*R*_int_	0.022
(sin θ/λ)_max_ (Å^−1^)	0.614

Refinement	
*R*[*F* ^2^ > 2σ(*F* ^2^)], *wR*(*F* ^2^), *S*	0.038, 0.102, 1.04
No. of reflections	2933
No. of parameters	194
No. of restraints	1
H-atom treatment	H atoms treated by a mixture of independent and constrained refinement
Δρ_max_, Δρ_min_ (e Å^−3^)	0.45, −0.20

Computer programs: *CrysAlis PRO* (Agilent, 2012 ▸), *SHELXT2014/4* (Sheldrick, 2015*a* ▸), *SHELXL2018/3* (Sheldrick, 2015*b* ▸), *ORTEP-3 for Windows* (Farrugia, 2012 ▸), *DIAMOND* (Brandenburg, 2006 ▸) and *publCIF* (Westrip, 2010 ▸).

## supplementary crystallographic information

### Crystal data

**Table:**

C_32_H_30_N_4_O_2_S_4_	*D*_x_ = 1.371 Mg m^−^^3^
*M**_r_* = 630.84	Cu *K*α radiation, λ = 1.54178 Å
Orthorhombic, *P**b**c**n*	Cell parameters from 4566 reflections
*a* = 15.4653 (4) Å	θ = 3.4–71.1°
*b* = 7.9639 (2) Å	µ = 3.15 mm^−^^1^
*c* = 24.8116 (7) Å	*T* = 100 K
*V* = 3055.90 (14) Å^3^	Plate, yellow
*Z* = 4	0.27 × 0.14 × 0.07 mm
*F*(000) = 1320	

### Data collection

**Table:**

Agilent Xcalibur, Eos, Gemini diffractometer	2933 independent reflections
Radiation source: Enhance (Cu) X-ray Source	2637 reflections with *I* > 2σ(*I*)
Graphite monochromator	*R*_int_ = 0.022
Detector resolution: 16.1952 pixels mm^-1^	θ_max_ = 71.3°, θ_min_ = 3.6°
ω scans	*h* = −18→18
Absorption correction: multi-scan (CrysAlisPro; Agilent, 2012)	*k* = −7→9
*T*_min_ = 0.819, *T*_max_ = 1.000	*l* = −29→30
10206 measured reflections	

### Refinement

**Table:**

Refinement on *F*^2^	Primary atom site location: dual
Least-squares matrix: full	Secondary atom site location: difference Fourier map
*R*[*F*^2^ > 2σ(*F*^2^)] = 0.038	Hydrogen site location: mixed
*wR*(*F*^2^) = 0.102	H atoms treated by a mixture of independent and constrained refinement
*S* = 1.04	*w* = 1/[σ^2^(*F*_o_^2^) + (0.0662*P*)^2^ + 1.2702*P*] where *P* = (*F*_o_^2^ + 2*F*_c_^2^)/3
2933 reflections	(Δ/σ)_max_ = 0.001
194 parameters	Δρ_max_ = 0.45 e Å^−^^3^
1 restraint	Δρ_min_ = −0.20 e Å^−^^3^

### Special details

**Table:**

Geometry. All esds (except the esd in the dihedral angle between two l.s. planes) are estimated using the full covariance matrix. The cell esds are taken into account individually in the estimation of esds in distances, angles and torsion angles; correlations between esds in cell parameters are only used when they are defined by crystal symmetry. An approximate (isotropic) treatment of cell esds is used for estimating esds involving l.s. planes.

### Fractional atomic coordinates and isotropic or equivalent isotropic displacement parameters (Å^2^)

**Table:**

	*x*	*y*	*z*	*U*_iso_*/*U*_eq_	
S1	0.44788 (3)	0.20945 (5)	0.77532 (2)	0.02123 (14)	
S2	0.41121 (3)	0.47744 (6)	0.69155 (2)	0.02165 (14)	
O1	0.32052 (8)	0.08476 (16)	0.89877 (5)	0.0239 (3)	
H1O	0.3312 (16)	0.141 (3)	0.8711 (7)	0.036*	
N1	0.30058 (9)	0.38409 (19)	0.76780 (6)	0.0210 (3)	
N2	0.28689 (10)	0.28083 (18)	0.81313 (6)	0.0200 (3)	
C1	0.37582 (11)	0.3625 (2)	0.74728 (7)	0.0188 (3)	
C2	0.20696 (11)	0.2752 (2)	0.82727 (7)	0.0208 (4)	
H2	0.166079	0.339978	0.807651	0.025*	
C3	0.17688 (11)	0.1739 (2)	0.87198 (7)	0.0202 (3)	
C4	0.23359 (11)	0.0816 (2)	0.90530 (7)	0.0197 (3)	
C5	0.19995 (12)	−0.0185 (2)	0.94637 (7)	0.0221 (4)	
H5	0.237862	−0.080587	0.968950	0.027*	
C6	0.11138 (13)	−0.0276 (2)	0.95434 (7)	0.0246 (4)	
H6	0.089111	−0.097226	0.982149	0.029*	
C7	0.05458 (11)	0.0638 (3)	0.92221 (7)	0.0259 (4)	
H7	−0.006038	0.057531	0.928079	0.031*	
C8	0.08769 (11)	0.1640 (2)	0.88161 (7)	0.0236 (4)	
H8	0.049188	0.227355	0.859785	0.028*	
C9	0.31721 (11)	0.6135 (2)	0.68128 (7)	0.0241 (4)	
H9A	0.268116	0.547317	0.666962	0.029*	
H9B	0.299350	0.664563	0.715912	0.029*	
C10	0.34232 (11)	0.7481 (2)	0.64183 (7)	0.0206 (4)	
C11	0.38760 (12)	0.8900 (2)	0.65887 (7)	0.0249 (4)	
H11	0.402651	0.901799	0.695798	0.030*	
C12	0.41082 (12)	1.0139 (2)	0.62245 (8)	0.0265 (4)	
H12	0.441428	1.109823	0.634869	0.032*	
C13	0.39015 (12)	1.0006 (2)	0.56792 (8)	0.0246 (4)	
C14	0.34520 (12)	0.8595 (2)	0.55108 (7)	0.0252 (4)	
H14	0.330262	0.848022	0.514124	0.030*	
C15	0.32150 (12)	0.7340 (2)	0.58738 (7)	0.0234 (4)	
H15	0.290918	0.638161	0.574895	0.028*	
C16	0.41484 (14)	1.1379 (3)	0.52889 (9)	0.0370 (5)	
H16A	0.408036	1.097062	0.491877	0.056*	
H16B	0.475189	1.170350	0.534944	0.056*	
H16C	0.377305	1.235519	0.534517	0.056*	

### Atomic displacement parameters (Å^2^)

**Table:**

	*U*^11^	*U*^22^	*U*^33^	*U*^12^	*U*^13^	*U*^23^
S1	0.0191 (2)	0.0229 (2)	0.0217 (2)	0.00008 (15)	0.00049 (14)	0.00357 (16)
S2	0.0204 (2)	0.0248 (2)	0.0197 (2)	0.00156 (16)	0.00321 (15)	0.00481 (16)
O1	0.0191 (6)	0.0272 (7)	0.0254 (6)	0.0013 (5)	0.0003 (5)	0.0051 (5)
N1	0.0216 (7)	0.0242 (7)	0.0173 (7)	−0.0009 (6)	−0.0002 (5)	0.0020 (6)
N2	0.0213 (7)	0.0223 (8)	0.0163 (7)	−0.0009 (6)	0.0004 (5)	0.0009 (5)
C1	0.0200 (8)	0.0198 (8)	0.0167 (8)	−0.0019 (6)	−0.0017 (6)	−0.0001 (6)
C2	0.0207 (8)	0.0227 (9)	0.0189 (8)	0.0017 (7)	−0.0024 (6)	−0.0021 (6)
C3	0.0224 (8)	0.0215 (8)	0.0168 (8)	−0.0005 (7)	0.0009 (6)	−0.0035 (7)
C4	0.0200 (8)	0.0199 (8)	0.0191 (8)	−0.0021 (7)	0.0011 (6)	−0.0045 (6)
C5	0.0266 (9)	0.0214 (9)	0.0182 (8)	−0.0001 (7)	−0.0008 (7)	−0.0015 (6)
C6	0.0299 (10)	0.0249 (9)	0.0189 (8)	−0.0053 (7)	0.0059 (7)	−0.0022 (7)
C7	0.0194 (9)	0.0343 (10)	0.0239 (9)	−0.0033 (7)	0.0029 (7)	−0.0041 (8)
C8	0.0204 (9)	0.0295 (9)	0.0211 (9)	0.0005 (7)	−0.0010 (6)	−0.0021 (7)
C9	0.0179 (8)	0.0283 (9)	0.0261 (9)	0.0034 (7)	0.0009 (7)	0.0069 (7)
C10	0.0170 (8)	0.0223 (8)	0.0226 (9)	0.0041 (6)	0.0009 (6)	0.0024 (7)
C11	0.0252 (9)	0.0282 (9)	0.0215 (9)	0.0031 (7)	−0.0033 (7)	−0.0024 (7)
C12	0.0234 (9)	0.0215 (9)	0.0347 (11)	−0.0020 (7)	−0.0059 (7)	−0.0020 (7)
C13	0.0205 (8)	0.0234 (9)	0.0299 (10)	0.0023 (7)	0.0004 (7)	0.0066 (7)
C14	0.0288 (9)	0.0275 (9)	0.0193 (8)	0.0027 (7)	−0.0031 (7)	0.0009 (7)
C15	0.0240 (9)	0.0208 (8)	0.0254 (9)	−0.0014 (7)	−0.0041 (7)	−0.0008 (7)
C16	0.0354 (11)	0.0336 (11)	0.0420 (12)	−0.0050 (9)	0.0003 (9)	0.0133 (9)

### Geometric parameters (Å, º)

**Table:**

S1—C1	1.7921 (17)	C7—H7	0.9500
S1—S1^i^	2.0439 (8)	C8—H8	0.9500
S2—C1	1.7463 (17)	C9—C10	1.503 (2)
S2—C9	1.8308 (18)	C9—H9A	0.9900
O1—C4	1.354 (2)	C9—H9B	0.9900
O1—H1O	0.839 (10)	C10—C15	1.393 (2)
N1—C1	1.282 (2)	C10—C11	1.395 (3)
N1—N2	1.409 (2)	C11—C12	1.385 (3)
N2—C2	1.286 (2)	C11—H11	0.9500
C2—C3	1.448 (2)	C12—C13	1.394 (3)
C2—H2	0.9500	C12—H12	0.9500
C3—C8	1.402 (2)	C13—C14	1.386 (3)
C3—C4	1.412 (2)	C13—C16	1.510 (3)
C4—C5	1.394 (2)	C14—C15	1.394 (3)
C5—C6	1.386 (3)	C14—H14	0.9500
C5—H5	0.9500	C15—H15	0.9500
C6—C7	1.392 (3)	C16—H16A	0.9800
C6—H6	0.9500	C16—H16B	0.9800
C7—C8	1.383 (3)	C16—H16C	0.9800
			
C1—S1—S1^i^	104.58 (6)	C10—C9—H9A	110.1
C1—S2—C9	99.87 (8)	S2—C9—H9A	110.1
C4—O1—H1O	107.7 (17)	C10—C9—H9B	110.1
C1—N1—N2	112.04 (14)	S2—C9—H9B	110.1
C2—N2—N1	112.49 (14)	H9A—C9—H9B	108.4
N1—C1—S2	121.92 (13)	C15—C10—C11	118.36 (16)
N1—C1—S1	120.10 (13)	C15—C10—C9	120.95 (16)
S2—C1—S1	117.98 (10)	C11—C10—C9	120.68 (16)
N2—C2—C3	122.48 (16)	C12—C11—C10	120.62 (17)
N2—C2—H2	118.8	C12—C11—H11	119.7
C3—C2—H2	118.8	C10—C11—H11	119.7
C8—C3—C4	118.81 (16)	C11—C12—C13	121.29 (17)
C8—C3—C2	118.54 (16)	C11—C12—H12	119.4
C4—C3—C2	122.63 (16)	C13—C12—H12	119.4
O1—C4—C5	117.93 (16)	C14—C13—C12	117.98 (17)
O1—C4—C3	122.47 (15)	C14—C13—C16	121.39 (18)
C5—C4—C3	119.59 (16)	C12—C13—C16	120.62 (18)
C6—C5—C4	120.20 (17)	C13—C14—C15	121.21 (17)
C6—C5—H5	119.9	C13—C14—H14	119.4
C4—C5—H5	119.9	C15—C14—H14	119.4
C5—C6—C7	120.98 (17)	C10—C15—C14	120.53 (17)
C5—C6—H6	119.5	C10—C15—H15	119.7
C7—C6—H6	119.5	C14—C15—H15	119.7
C8—C7—C6	119.01 (17)	C13—C16—H16A	109.5
C8—C7—H7	120.5	C13—C16—H16B	109.5
C6—C7—H7	120.5	H16A—C16—H16B	109.5
C7—C8—C3	121.39 (17)	C13—C16—H16C	109.5
C7—C8—H8	119.3	H16A—C16—H16C	109.5
C3—C8—H8	119.3	H16B—C16—H16C	109.5
C10—C9—S2	107.91 (12)		
			
C1—N1—N2—C2	−166.57 (15)	C5—C6—C7—C8	0.4 (3)
N2—N1—C1—S2	−178.60 (11)	C6—C7—C8—C3	0.6 (3)
N2—N1—C1—S1	2.0 (2)	C4—C3—C8—C7	−1.2 (3)
C9—S2—C1—N1	2.05 (17)	C2—C3—C8—C7	177.24 (16)
C9—S2—C1—S1	−178.53 (10)	C1—S2—C9—C10	168.30 (13)
S1^i^—S1—C1—N1	174.92 (13)	S2—C9—C10—C15	97.92 (17)
S1^i^—S1—C1—S2	−4.51 (11)	S2—C9—C10—C11	−81.68 (18)
N1—N2—C2—C3	178.45 (15)	C15—C10—C11—C12	0.3 (3)
N2—C2—C3—C8	−174.53 (17)	C9—C10—C11—C12	179.90 (16)
N2—C2—C3—C4	3.8 (3)	C10—C11—C12—C13	−0.3 (3)
C8—C3—C4—O1	−179.81 (15)	C11—C12—C13—C14	0.2 (3)
C2—C3—C4—O1	1.8 (3)	C11—C12—C13—C16	179.13 (18)
C8—C3—C4—C5	0.8 (3)	C12—C13—C14—C15	−0.2 (3)
C2—C3—C4—C5	−177.54 (16)	C16—C13—C14—C15	−179.12 (18)
O1—C4—C5—C6	−179.26 (15)	C11—C10—C15—C14	−0.3 (3)
C3—C4—C5—C6	0.2 (3)	C9—C10—C15—C14	−179.90 (16)
C4—C5—C6—C7	−0.8 (3)	C13—C14—C15—C10	0.3 (3)

Symmetry code: (i) −*x*+1, *y*, −*z*+3/2.

### Hydrogen-bond geometry (Å, º)

*Cg*1 is the centroid of the (C10–C15) ring.

**Table:**

*D*—H···*A*	*D*—H	H···*A*	*D*···*A*	*D*—H···*A*
O1—H1*O*···N2	0.84 (2)	1.94 (2)	2.6877 (19)	148 (2)
C9—H9*A*···*Cg*1^ii^	0.99	2.93	3.9075 (18)	169

Symmetry code: (ii) *x*, −*y*−1, *z*−1/2.
